# Palatal myoclonus and hypertrophic olivary degeneration following wernekinck commissure syndrome: a case report

**DOI:** 10.1186/s12883-023-03157-y

**Published:** 2023-03-29

**Authors:** Qian Zhang, Jiahuan Guo, Xingquan Zhao, Xinghu Zhang, Yuetao Ma

**Affiliations:** 1 Department of Neurology, Beijing Tiantan Hospital, Capital Medical University, Beijing, China; 2 China National Clinical Research Center for Neurological Diseases, Beijing, China; 3 Research Unit of Artificial Intelligence in Cerebrovascular Disease, Chinese Academy of Medical Sciences, Beijing, China; 4 Center of Stroke, Beijing Institute of Brain Disorders, Capital Medical University, Beijing, China

**Keywords:** Hypertrophic olivary degeneration, Palatal myoclonus, Wernekinck Commissure Syndrome

## Abstract

**Background:**

Hypertrophic olivary degeneration (HOD), a rare form of transsynaptic degeneration, is secondary to dentato-rubro-olivary pathway injuries in some cases. We describe a unique case of an HOD patient who presented with palatal myoclonus secondary to Wernekinck commissure syndrome caused by a rare bilateral “heart-shaped” infarct lesion in the midbrain.

**Case presentation:**

A 49-year-old man presented with progressive gait instability in the past 7 months. The patient had a history of posterior circulation ischemic stroke presenting with diplopia, slurred speech, and difficulty in swallowing and walking 3 years prior to admission. The symptoms improved after treatment. The feeling of imbalance appeared and was aggravated gradually in the past 7 months. Neurological examination demonstrated dysarthria, horizontal nystagmus, bilateral cerebellar ataxia, and 2–3 Hz rhythmic contractions of the soft palate and upper larynx. Magnetic resonance imaging (MRI) of the brain performed 3 years prior to this admission showed an acute midline lesion in the midbrain exhibiting a remarkable “heart appearance” on diffusion weighted imaging. MRI after this admission revealed T2 and FLAIR hyperintensity with hypertrophy of the bilateral inferior olivary nucleus. We considered a diagnosis of HOD resulting from a midbrain heart-shaped infarction, which caused Wernekinck commissure syndrome 3 years prior to admission and later HOD. Adamantanamine and B vitamins were administered for neurotrophic treatment. Rehabilitation training was also performed. One year later, the symptoms of this patient were neither improved nor aggravated.

**Conclusion:**

This case report suggests that patients with a history of midbrain injury, especially Wernekinck commissure injury, should be alert to the possibility of delayed bilateral HOD when new symptoms occur or original symptoms are aggravated.

**Supplementary Information:**

The online version contains supplementary material available at 10.1186/s12883-023-03157-y.

## Background

Hypertrophic olivary degeneration (HOD) is a rare form of transsynaptic degeneration due to damage to the dentato-rubro-olivary pathway and mitochondrial dysfunction [1]. HOD has characteristic presentations, including palatal myoclonus and nystagmus. The etiology can be found in most cases of unilateral HOD. In contrast, no obvious cause was found in more than half of the bilateral cases [2, 3].

Wernekinck commissure syndrome is a rare midbrain syndrome characterized by cerebellar ataxia, ophthalmoplegia, and palatal myoclonus [4]. To date, very few bilateral midbrain infarctions characterized by a “heart-shaped lesion” have been reported. Herein, we present a typical case of bilateral HOD following Wernekinck commissure syndrome caused by a bilateral “heart-shaped lesion”. Written informed consent was obtained from the patient.

## Case presentation

A 49-year-old man suffered from posterior circulation ischemic stroke presenting with diplopia, slurred speech, and difficulty swallowing and walking 3 years prior to admission. Only mild dysarthria and unsteady gait remained after weeks of treatment and physical rehabilitation, which did not affect his daily life. More than two years after the ischemic stroke event, the patient developed a feeling of aggravated imbalance and walking instability. The symptoms aggravated gradually in the past 7 months, and he was not able to run or ascend stairs independently on admission. The patient had no other medical history or regular medication use. He had smoked 8 cigarettes a day for 40 years and stopped smoking for 3 years. No significant family history was recorded.

Neurological examination demonstrated dysarthria, horizontal nystagmus, bilateral cerebellar ataxia, and 2–3 Hz rhythmic contractions of the soft palate and upper larynx **(Video)**. His gait was clumsy.

**Fig. 1:**
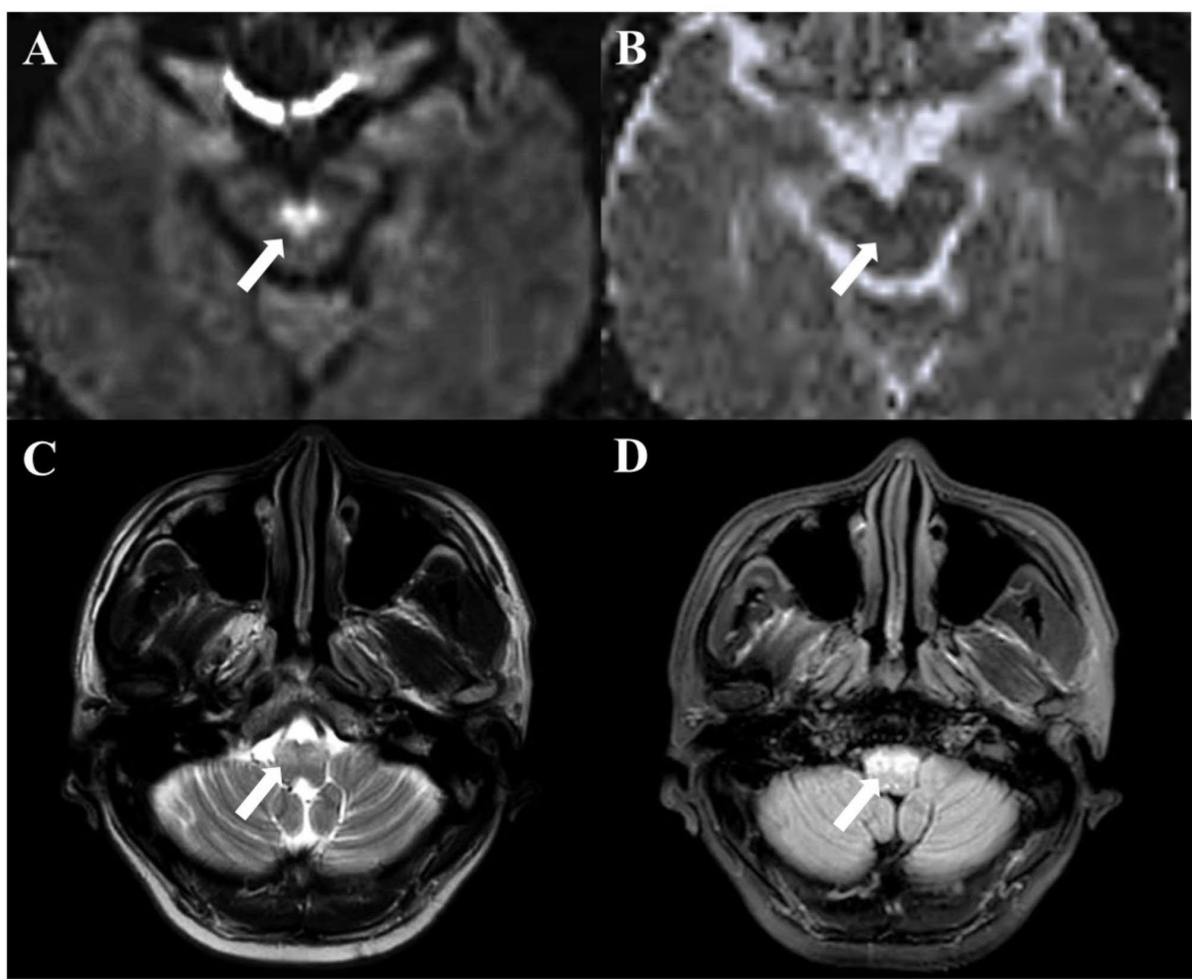
Brain magnetic resonance imaging axial sections. (A) Diffusion weighted imaging and (B) apparent diffusion coefficient sequences showed diffusion restriction in the caudal midbrain region 3 years prior to this admission. (C) T2-weighed and (D) fluid-attenuated inversion recovery images showed bilateral swollen and hyperintense olives during this admission

The routine laboratory examination (including autoantibody spectrum, paraneoplastic antibodies, vitamin B12, folate, etc.) revealed no specific abnormalities. Cerebrospinal fluid examination was normal. The results of pressure, cell counts, protein, glucose, oligoclonal band, immunoglobulin g index, AQP4, MOG and MBP antibody were all negative or normal. 1.5T magnetic resonance imaging (MRI) of the brain (Fig. 1A and B) performed approximately 10 days after ischemic stroke onset 3 years prior to this admission showed an acute midline lesion in the midbrain exhibiting a remarkable “heart appearance” on diffusion weighted imaging. 3T-MRI after this admission revealed T2 and FLAIR hyperintensity with hypertrophy of the bilateral inferior olivary nucleus, and no other abnormalities were detected (Fig. 1C and D).

The patient was finally diagnosed with bilateral HOD resulting from a midbrain heart-shaped infarction and Wernekinck commissure syndrome 3 years prior to admission. Adamantanamine 100 mg/day was administered to improve motor function, and B vitamins were administered for neurotrophic treatment. Rehabilitation training was also performed. The medication was discontinued by the patient after discharge, and rehabilitation training was also discontinued due to the COVID-19 pandemic. One year later, the symptoms of this patient neither improved nor were aggravated. We suggested this patient continue rehabilitation.

## Discussion and conclusion

HOD is a rare form of multisynaptic and transneuronal degeneration of the inferior olivary nucleus, which usually develops secondary to the impairment of the fibers in the Guillain–Mollaret triangle [5]. The oliva is located in the anterior lateral medulla and participates in the integration of posture and motion. It receives fibers from the ipsilateral red nucleus in the mesencephalon, and the efferent fibers from the oliva enter the contralateral dentate nucleus in the cerebellum. Then, cerebellorubral fibers from the dentate nucleus connect the contralateral red nucleus. These anatomic structures and fibers are also referred to as the dentato-rubro-olivary pathway [6]. Impairment of this pathway is considered to be the pathological basis of HOD [7].

A variety of underlying etiologies have been described, including vascular lesions, toxicity, trauma, surgery, vascular malformation and tumors [5]. HOD may occur several months or even years in some cases after the primary cause. Studies have reported that HOD is almost predominantly unilateral [8]. Bilateral HOD is rare and can be caused by a para-median pontine lesion that affects both the central tegmental tract and the superior cerebellar peduncle and leads to inferior olivary nucleus degeneration [9]. Wernekinck commissure syndrome is a typical but rare syndrome caused by a lesion in the Wernekinck commissure, characterized by bilateral cerebellar dysfunction, variable eye-movement disorders, and occasionally delayed palatal myoclonus [10]. Bilateral caudal paramedian midbrain infarction demonstrated the characteristic “heart appearance” on MRI and clinically demonstrated Wernekinck commissure syndrome was first reported in 2017 [11]. Delayed palatal myoclonus and hypertrophic olivary degeneration in the medulla oblongata were caused by primary lesions in the dento-rubro-olivary pathway. In a previous case report, Mossuto-Agatiello [12] reported five patients with unilateral caudal paramedian midbrain lesions, all the patients showed delayed HOD and only one patient showed palatal tremor. Theoretically, the midline lesion located at the decussation of the superior cerebellar peduncle in Wernekinck commissure syndrome can lead to bilateral HOD due to injury to the bilateral dentate-olivary fibers as shown by Wang [5]. Liu [13] reported two Wernekinck commissure syndrome cases but neither HOD nor palatal tremor was found. One explanation was that the disease course and follow-up time were short in those two cases. In one case, Zhou [14] reported HOD with palatal tremor in a patient with Wernekinck commissure syndrome after 3 months. The interval between the primary injury and HOD varied considerably. HOD was reported within a few months of the primary injury in some cases, while there were also HOD cases that occurred years after the primary injury [2, 5]. In the present case, we report a unique case of delayed bilateral HOD that occurred more than two years after Wernekinck comnnissure syndrome with a bilateral heart-shaped bilateral midbrain lesion. There was no clinical or imaging evidence of other causes of HOD during these years, and bilateral HOD was confirmed by MRI after admission. Therefore, the bilateral midbrain infarction was thought to be the cause of HOD at this time.

The clinical signs of HOD include palatal myoclonus, ocular myoclonus, nystagmus, Holmes tremor, symptoms of cerebellar and brainstem dysfunction such as dizziness, diplopia and blurred vision during or after the improvement of the primary disease. Among them, symptomatic palatal myoclonus is a hallmark symptom of HOD that often arises from lesions localizing in the Guillain–Mollaret triangle and loss of inhibitory inputs into the olivary nucleus. Symptomatic palatal myoclonus refers to rhythmic 1–4 Hz involuntary movements of the soft palate produced by irregular contractions of agonist muscles [15].

Histological changes in HOD include vacuolar degeneration, neuronal and astrocytic hypertrophy and gliosis. The disease course of HOD is a dynamic and evolving process that may take months to years to progress. The evolution of HOD may include olivary degeneration, neuronal olivary hypertrophy, olivary enlargement, degeneration of neurons and atrophy of the olivary nucleus [5]. A previous imaging study showed that visible changes on MRI correlate well with the described sequential histopathologic findings [16]. In the first 6 months of the ictus, an increased T2 signal in the inferior olivary nucleus can be seen without hypertrophy. Then, hypertrophy of the inferior olivary nucleus appears and may last 3–4 years. Finally, olivary shrinkage becomes apparent, and increased T2 signals persist.

To date, there are no available specific therapies for patients with HOD. Previous studies have demonstrated that clonazepam, levodopa, dopaminergic agents, levetiracetam and deep brain stimulation may have varying degrees of efficacy [17]. HOD is considered to be a self-limiting disease, and the prognosis of HOD depends on the etiology. Unilateral HOD usually has a better and faster recovery of symptoms than bilateral HOD [9]. In our case, the main symptom that had an adverse impact on quality of life was gait instability; therefore, adamantanamine was administered for treatment. However, adamantanamine treatment was stopped due to inefficacy.

In conclusion, bilateral HOD is a rare degeneration that is mainly secondary to diseases affecting the Guillain–Mollaret triangle. In clinical practice, the diagnosis of HOD is not always recognized and may be mistaken for a second stroke or other pathology [18]. Increased awareness of patients at risk and pathophysiological mechanisms is essential for clinical surveillance and disease management. Our case suggests that patients with a history of midbrain injury, especially Wernekinck commissure, should be alert to the possibility of delayed bilateral HOD when new symptoms occur or original symptoms are aggravated.

## Electronic supplementary material

Below is the link to the electronic supplementary material.

Supplementary Material 1

Supplementary Material 2

## Data Availability

Not applicable.

## Abbreviations

HOD: Hypertrophic olivary degeneration
MRI: Magnetic resonance imaging
