# Supplementary material for: Palatal myoclonus and hypertrophic olivary degeneration following wernekinck commissure syndrome: a case report
Source: BMC Neurol. 2023 Mar 29;23:127. doi: 10.1186/s12883-023-03157-y (PMC10050798; doi:10.1186/s12883-023-03157-y)
Supplement: Supplementary file 2 — Supplementary Material 2 [file 12883_2023_3157_MOESM2_ESM.docx]

**Video legend**

**Video.** Palatal myoclonus of the patient.
